# Solid locked intramedullary nailing for expeditious return of bone-setting-induced abnormal fracture union victims to work in South-western Nigeria

**DOI:** 10.1038/s41598-022-25862-3

**Published:** 2022-12-12

**Authors:** Stephen Adesope Adesina, Samuel Uwale Eyesan, Isaac Olusayo Amole, Akinsola Idowu Akinwumi, Olufemi Timothy Awotunde, Adewumi Ojeniyi Durodola, James Idowu Owolabi

**Affiliations:** 1 Department of Family Medicine, Bowen University Teaching Hospital, P. O. Box 15, Ogbomoso, Oyo State Nigeria; 2 Department of Family Medicine, Bowen University, P.M.B 284, Iwo, Osun State Nigeria; 3 Department Surgery, Bowen University Teaching Hospital, P. O. Box 15, Ogbomoso, Oyo State Nigeria; 4 Department Surgery, Bowen University, P.M.B 284, Iwo, Osun State Nigeria; 5 Department of Family Medicine, Afe Babalola University, km 8.5, Afe Babalola Way, Ado-Ekiti, Ekiti State Nigeria

**Keywords:** Health care, Medical research

## Abstract

Wage earning in low- and middle-income countries (LMICs) is predominantly through physical labour. Consequently, limb-related disabilities caused by abnormal fracture unions (AFUs) preclude gainful employment and perpetuate the cycle of poverty. Many AFUs result from traditional bone-setting (TBS), a pervasive treatment for long bone fractures in LMICs. The objective of this study was to accentuate the expediency of solid locked intramedullary nail in the early restoration of victims of TBS-induced abnormal fracture unions (AFUs) to their pre-injury functioning, including work. One hundred AFUs in 98 patients treated with a solid locked intramedullary nail in our center over a period of 7 years were prospectively studied. We found the mean age to be 47.97 years. Males constituted 63.9% of the patients’ population. Atrophic non-union accounted for 54.1% of the AFUs. The mean fracture-surgery interval was 21.30 months. By the 12th post-operative week, more than 75% of the fractures had achieved knee flexion/shoulder abduction beyond 90°, were able to squat and smile (or do shoulder abduction-external rotation), and were able to bear weight fully. The study demonstrated the expediency of solid locked nail in salvaging TBS-induced abnormal fracture unions in a way that permitted early return to pre-injury daily activities and work, thereby reducing fracture-associated poverty.

## Introduction

Socioeconomic functioning in low- and middle-income countries (LMICs) often requires a good ambulatory capacity and satisfactory use of the upper limbs since wage earning is predominantly through physical labour. Hence, limb-related disabilities render an individual incapable of gainful employment and perpetuate the cycle of poverty^1^. Therefore, poverty-mitigating fracture care in LMICs must allow early use of the limbs to foster prompt return to pre-injury daily activities and work. This is particularly desirable in victims of abnormal fracture unions (AFUs) whose disability has often resulted in psychological distress, social and economic costs in terms of loss of work days and eventual higher cost of effective treatment^2–4^. A faster functional recovery and return to work which can lessen the economic impact of an injury requires early weight bearing (WB)^5^. In high income countries, various newer strategies that ensure quicker restoration of function are now available to treat AFUs but these are currently non-existent in most LMICs^6–8^. Thus, most surgeons in LMICs have continued to treat AFUs with methods which are less compatible with early WB. These include external fixation, and internal fixation using plates or unlocked intramedullary (IM) nails^9–12^.

Long bone fractures have been turned into an epidemic by rapid urbanizations, increased use of motorized vehicles and incessant violent incidents in many LMICs. Regrettably, traditional bone-setting (TBS) has continued to flourish in LMICs as the first line (or the only) care for these injuries. This is due to superstitious beliefs, ignorance, cultural norms, poor orthodox health system and high cost of modern fracture care facilities^2,9,13,14^. Traditional bonesetters are found in most communities of developing countries^13,14,16^. These are unorthodox practitioners without any training in a formal, medical education setting, having inherited their purported skills from older generations in the family^15,16^. While a few previous authors identified some usefulness of TBS^13,17^, most other studies have established havocs done to the injured by bonesetters^18,19^. Limb gangrene, tetanus, chronic bone infection or eventual fatality are the most devastating disasters of TBS^18,19^. However, also associated with the practice is disability caused by AFUs, including delayed union, mal-union and non-union^10,20^.

Controversies exist about the standard definition but the term ‘non-union’ has recently been applied to a fracture which shows no progressive signs of healing after three months of treatment^3,6,21,22^. Atrophic non-union means a fracture site is devoid of healing potential with accompanying dearth of callus, usually as a result of biological factors. The fracture has only minimal amounts of callus in oligotrophic non-unions due to inadequate immobilisation while hypertrophic non-union is characterized by exuberant callus formation but in a disorganized manner, due to inadequate mechanical stability^3^. Mal-union describes a fracture that healed non-anatomically with resultant length, alignment or angular deformities and, often functional impairment^10^. Delayed union applies to a fracture which in spite of progressing towards union, has not healed in the expected amount of time for a comparable fracture^22^.

In this study, we present the data on patients who had their TBS-induced AFUs fixed with the Surgical Implant Generation Network’s (SIGN) locked IM nail in our centre. The aim is to accentuate the expediency of solid locked IM nail in early restoration of victims of TBS AFUs in LMICs to their pre-injury functioning, including work.

## Methods

### Study site

The study was carried out at Bowen University Teaching Hospital, Ogbomoso, a semi-urban city located in South-Western Nigeria. The city is inhabited by artisans, civil servants, subsistence farmers and small business owners. The hospital serves other nearby villages/towns composed of similar populations.

### Study design

Over a period of 7 years (July 2014 to June 2021), we used the SIGN locked IM nail to treat a total of 100 fractures of the humerus, femur and tibia in 98 patients. All the fractures were TBS-induced AFUs. Using a descriptive study design, data were collected prospectively on all the fractures. The data included patient and fracture characteristics, as well as treatment outcome. The data were analysed with SPSS version 23 (IBM Corp, New York, USA) and presented as descriptive statistics in tables and figures.

The standard occupational classification system designed by the Office of Population Census and Surveys, London (OPCS 1991)^23^ and modified for Nigeria^24^ was used to classify the patients into occupational classes 1 to 3 as follows:

- Class 1—Skilled workers e.g. professionals and managerial officers and retirees of this cadre.
- Class 2—Unskilled workers e.g. artisans and traders.
- Class 3—Dependants. e.g. retirees of class 2, those not on pensions, house wives of class 2 cadre, students.

Fracture location and morphology were defined according to AO/OTA guideline^25^. Abnormal unions were grouped into atrophic non-union (≥ 3 months, scanty or no callus on plain radiograph, motion at fracture site)^3,6,22^, hypertrophic non-union (≥ 3 months, excessive callus on plain radiograph, motion at fracture site)^3,6,22^, mal-union (healed but mal-aligned or shortened)^10^, and delayed union (< 3 months, no clinical or radiographic evidence of ongoing healing)^22^. The time length between the occurrence of fracture and performance of surgery (*fracture-surgery interval)* was grouped into: ≤ 3 months, > 3 but ≤ 6 months, > 6 but ≤ 9 months, > 9 but ≤ 18 months, and > 18 months. The time length between skin incision and closure (*duration of surgery*) was categorized into: within 1 h, within 2 h, within 3 h, within 4 h, and > 4 h (Table 2).

### Operative and post-operative care

Following satisfactory routine pre-operative work-up, each patient underwent a one-stage surgical fixation of his/her fractures with the SIGN nail. After anaesthesia was given, ankylosed limb joints were manipulated to improve the range of motion. All of the fractures had open reduction. The fibrous tissues were excised in non- and delayed unions while mal-unions were osteotomized. The bone canals were opened up with bone curette and manual reamers.

Reduction was achieved manually either by gradually distracting the fragments with a periosteal elevator placed between them or by hooking the fragments together in flexion while gradually extending the fracture site. Rotational malalignment was avoided by stabilizing the reduced fracture using a Lowman Clamp while aligning the linea aspera of the femur, or anterior border of the tibia. For the humerus, limb was placed in anatomical position beside the patient’s body. Subsequently, the locked nail was inserted as described by the manufacturer^26^. Autologous bone grafting was done for non-unions. The graft was harvested from the proximal tibia. All the patients had a five-day course of intravenous ceftriaxone. Pre- and post-operative radiographs were taken.

As permitted by their fracture pattern, bone integrity, and general condition, the patients were ambulated from the first post-operative day, and encouraged to move their joints. They were discharged from the hospital in the first or second post-operative week. Follow-up was continued at the out-patient clinic with plain radiographs and a test of ability to ‘squat and smile’ (femur and tibia) or do shoulder abduction-external rotation (humerus). The follow-ups were done at least twice—at six weeks and 12 weeks—but also at six and 12 months if painless ambulation or fracture healing was not achieved at the 12th week follow-up. The time taken to achieve full WB and knee flexion/shoulder abduction beyond 90° was noted. Occurrence of nerve palsy, presence of infection or need for a repeat surgery were also noted.

### Ethical consideration

All patients gave informed consent to be included in the study. All methods were carried out in accordance with relevant guidelines and regulations, and the study protocols were approved by Bowen University Teaching Hospital Research Ethics Committee.

## Results

**Figure 1:**
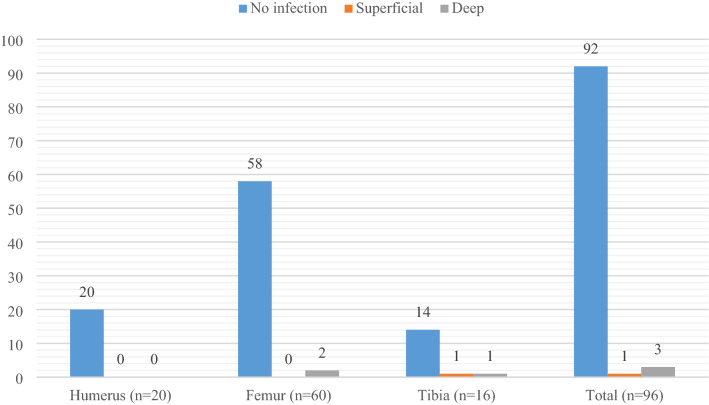
Infection types.

**Figure 2:**
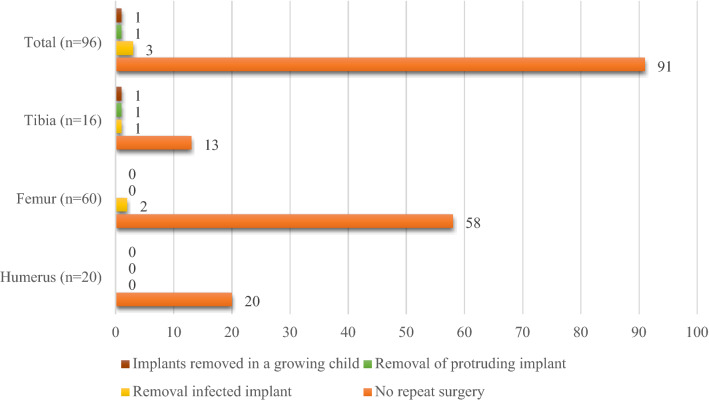
Type of repeat surgery done.

Over the study period, a total of 100 TBS-induced AFUs were treated in 98 patients. These included the 20 humerus, 64 femur and 16 tibia fractures. Of this, 96 fractures were seen for follow-up and were included in the analysis of treatment outcome (Table 4, Figs. 1 and 2), giving a follow-up rate of 96%. The four (4) femur fracture cases that were lost to follow-up were excluded. Three (15%) of the humerus fractures had iatrogenic radial nerve palsy which had recovered by the 12th week follow-up.

Table 1 shows the mean and range were 48.16 and 10–99 years respectively. Males constituted almost two-thirds (63.3%) of the patients’ population. Less than one-quarter (23.5%) were educated to tertiary level and only 17.3% belonged to occupational class 1. Motorcycle accidents was responsible for the highest proportion (40.8%) of the fractures.

**Table 1:** Patients' sociodemographic characteristics and cause of fracture.

Variables (n = 98)		Humerus (n = 20)	Femur (n = 63)	Tibia (n = 15)	Total (n = 98)
Age group (years) Mean age = 48.16 years; Age range = 10–99 years	10–19	0 (0.0)	2 (3.2)	1 (6.7)	3 (3.1)
	20–29	3 (15.0)	8 (12.7)	1 (6.7)	12 (12.2)
	30–39	4 (20.0)	10 (15.9)	2 (13.3)	16 (16.3)
	40–49	4 (20.0)	17 (27.0)	7 (46.7)	28 (28.6)
	50–59	5 (25.0)	5 (7.9)	2 (13.3)	12 (12.2)
	60–69	1 (5.0)	8 (12.7)	1 (6.7)	10 (10.2)
	70–79	3 (15.0)	9 (14.3)	1 (6.7)	13 (13.3)
	80–89	0 (0.0)	3 (4.8)	0 (0.0)	3 (3.1)
	90–99	0 (0.0)	1 (1.6)	0 (0.0)	1 (1.0)
Gender	Male	10 (50.0)	43 (68.3)	9 (60.0)	62 (63.3)
	Female	10 (50.0)	20 (31.7)	6 (40.0)	36 (36.7)
Marital status	Single	3 (15.0)	11 (17.5)	3 (20.0)	17 (17.3)
	Married	11 (55.0)	39 (61.9)	7 (46.7)	57 (58.2)
	Separated/Divorced	2 (10.0)	4 (6.3)	2 (13.3)	8 (8.2)
	Widowed	4 (20.0)	9 (14.3)	3 (20.0)	16 (16.3)
Education	None	4 (20.0)	14 (22.2)	2 (13.3)	20 (20.4)
	Primary	4 (20.0)	15 (23.8)	3 (20.0)	22 (22.4)
	Secondary	6 (30.0)	20 (31.7)	7 (46.7)	33 (33.7)
	Tertiary	6 (30.0)	14 (22.2)	3 (20.0)	23 (23.5)
Occupational class	Class 1	4 (20.0)	9 (14.3)	4 (26.7)	17 (17.3)
	Class 2	11 (55.0)	37 (58.7)	8 (53.3)	56 (57.2)
	Class 3	5 (25.0)	17 (27.0)	3 (20.0)	25 (24.5)
Cause of fracture	Motorcycle accident	8 (40.0)	26 (41.3)	6 (40.0)	40 (40.8)
	Motor vehicle accident	5 (25.0)	8 (12.7)	4 (26.7)	17 (17.3)
	Pedestrian injury	4 (20.0)	11 (17.5)	3 (20.0)	18 (18.4)
	Fall	2 (10.0)	18 (28.6)	2 (13.3)	22 (22.5)
	Assault	1 (5.0)	0 (0.0)	0 (0.0)	1 (1.0)

Table 2 reveals that most of humerus fractures were simple diaphyseal fractures with the simple transverse (12-A3) having the largest percentage (40.0%). The femur fractures were more of diaphyseal simple transverse (23.4%), intact wedge (20.3%) or simple oblique (17.2%). The tibia fractures were mostly diaphyseal simple transverse (37.5%) and simple oblique (32.2%) fractures.

**Table 2:** AO/OTA classification of the fractures.

Bone	AO/OTA classes	n (%)
Humerus (n = 20)	12-A1	3 (15.0)
	12-A2	4 (20.0)
	12-A3	8 (40.0)
	12-B2	1 (5.0)
	12-B3	1 (5.0)
	12-C3	2 (10.0)
	13-A3	1 (5.0)
Femur (n = 64)	31-A3	7 (10.9)
	32-A1	8 (12.5)
	32-A2	11 (17.2)
	32-A3	15 (23.4)
	32-B2	13 (20.3)
	32-B3	3 (4.7)
	32-C2	3 (4.7)
	32-C3	3 (4.7)
	33-A2	1 (1.6)
Tibia (n = 16)	42-A2	5 (31.2)
	42-A3	6 (37.5)
	42-B2	2 (12.5)
	42-C2	1 (6.3)
	42-C3	1 (6.3)
	43-A1	1 (6.3)

In Table 3, it is observed that there were more fractures on the right limb (55.0%) than the left (45.0%). Diaphyseal fractures constituted the highest proportion (90.0%). More than one-half (55.0%) of all the fractures were atrophic non-unions. The mean fracture-surgery interval was 21.04 months and surgery was completed within three hours in most of the cases.

**Table 3:** Fracture characteristics and treatment details.

Variable (n = 100)		Humerus (n = 20) n (%)	Femur (n = 64) n (%)	Tibia (n = 16) n (%)	Total (n = 100) n (%)
Side	Right	15 (75.0)	33 (51.6)	7 (43.8)	55 (55.0)
	Left	5 (25.0)	31 (48.4)	9 (56.3)	45 (45.0)
Location	Proximal end segment	0 (0.0)	7 (10.9)	0 (0.0)	7 (7.0)
	Diaphyseal segment	19 (95.0)	56 (87.5)	15 (93.7)	90 (90.0)
	Distal end segment	1 (5.0)	1 (1.6)	1 (6.3)	3 (3.0)
Type	Closed	20 (100.0)	60 (93.8)	13 (81.3)	93 (93.0)
	Initial open fracture but wound had healed	0 (0.0)	4 (6.3)	3 (18.7)	7 (7.0)
Abnormal union type	Atrophic non-union	19 (95.0)	33 (51.5)	3 (18.7)	55 (55.0)
	Hypertrophic non-union	1 (5.0)	22 (34.4)	10 (62.5)	33 (33.0)
	Mal-union	0 (0.0)	3 (4.7)	1 (6.3)	4 (4.0)
	Delayed union	0 (0.0)	6 (9.4)	2 (12.5)	8 (8.0)
Fracture-surgery interval (months) Mean = 21.04 Range = 1.12–219.03	≤ 3 months	0 (0.0)	6 (9.4)	2 (12.5)	8 (8.0)
	> 3 but ≤ 6 months	4 (20.0)	16 (25.0)	4 (25.0)	24 (24.0)
	> 6 but ≤ 9 months	2 (10.0)	10 (15.6)	2 (12.5)	14 (14.0)
	> 9 but ≤ 18 months	7 (35.0)	14 (21.9)	5 (31.3)	26 (26.0)
	> 18 months	7 (35.0)	18 (28.1)	3 (18.7)	28 (28.0)
Duration of surgery Mean = 2.10 h Range = 0.92–4 h	Within 1 h	1 (5.0)	1 (1.6)	2 (12.5)	4 (4.0)
	Within 2 h	9 (45.0)	24 (37.5)	12 (75.0)	45 (45.0)
	Within 3 h	10 (50.0)	33 (51.5)	2 (12.5)	45 (45.0)
	Within 4 h	0 (0.0)	6 (9.4)	0 (0.0)	6 (6.0)

In Table 4, it is observed that by the 12th post-operative week, 75% or more of the fractures had achieved knee flexion/shoulder abduction beyond 90°, were able to squat and smile (or do shoulder abduction-external rotation), had evidence of ongoing radiographic healing and were able to bear weight fully.

**Table 4:** Treatment outcome.

Variable (n = 96)		Humerus (n = 20) n (%)	Femur (n = 60) n (%)	Tibia (n = 16) n (%)	Total (n = 96) n (%)	Cumulative total (%)
Knee flexion (or shoulder abduction) > 90° noted at:	6-week follow-up	9 (45.0)	32 (53.3)	13 (81.2)	54 (56.3)	56.3
	12-week follow-up	6 (30.0)	16 (26.7)	2 (12.5)	24 (25.0)	81.3
	6-month follow-up	3 (15.0)	4 (6.7)	0 (0.0)	7 (7.3)	88.6
	Beyond 6-month follow-up	0 (0.0)	1 (1.7)	0 (0.0)	1 (1.0)	89.6
	Not achieved	1 (5.0)	2 (3.3)	0 (0.0)	3 (3.1)	92.7
	Stiff before surgery	1 (5.0)	5 (8.3)	1 (6.3)	7 (7.3)	100.0
Ability to squat and smile (or do shoulder abduction-external rotation noted at:	6-week follow-up	7 (35.0)	17 (28.3)	7 (43.7)	31 (32.3)	32.3
	12-week follow-up	9 (45.0)	26 (43.3)	6 (37.5)	41 (42.7)	75.0
	6-month follow-up	2 (10.0)	8 (13,3)	0 (0.0)	10 (10.4)	85.4
	Beyond 6-month follow-up	0 (0.0)	2 (3.3)	1 (6.3)	3 (3.1)	88.5
	Not achieved	1 (5.0)	3 (5.0)	1 (6.3)	6 (6.3)	94.8
	Stiff before surgery	1 (5.0)	4 (6.7)	1 (6.3)	5 (5.2)	100.0
Ongoing healing noted on radiograph at:	6-week follow-up	10 (50.0)	31 (51.7)	8 (50.0)	49 (51.0)	51.0
	12-week follow-up	8 (40.0)	24 (40.0)	8 (50.0)	40 (41.7)	92.7
	6-month follow-up	1 (5.0)	4 (6.7)	0 (0.0)	5 (5.2)	97.9
	Not achieved	1 (5.0)	1 (1.7)	0 (0.0)	2 (2.1)	100.0
Full weight bearing noted at:	6-week follow-up	9 (45.0)	26 (43.3)	7 (43.8)	42 (43.7)	43.7
	12-week follow-up	10 (50.0)	23 (38.3)	7 (43.8)	40 (41.7)	85.4
	6-month follow-up	1 (5.0)	11 (18.3)	2 (12.5)	14 (14.6)	100.0

Figure 1 shows four cases got infected, including 3 deep and 1 superficial infection of the surgical site, giving an overall infection rate of 4.1%.

Figure 2 shows five (5.2%) patients had their implants removed: three because of deep infection, one because the implant was protruding into a joint, and one in a growing child.

**Figure 3:**
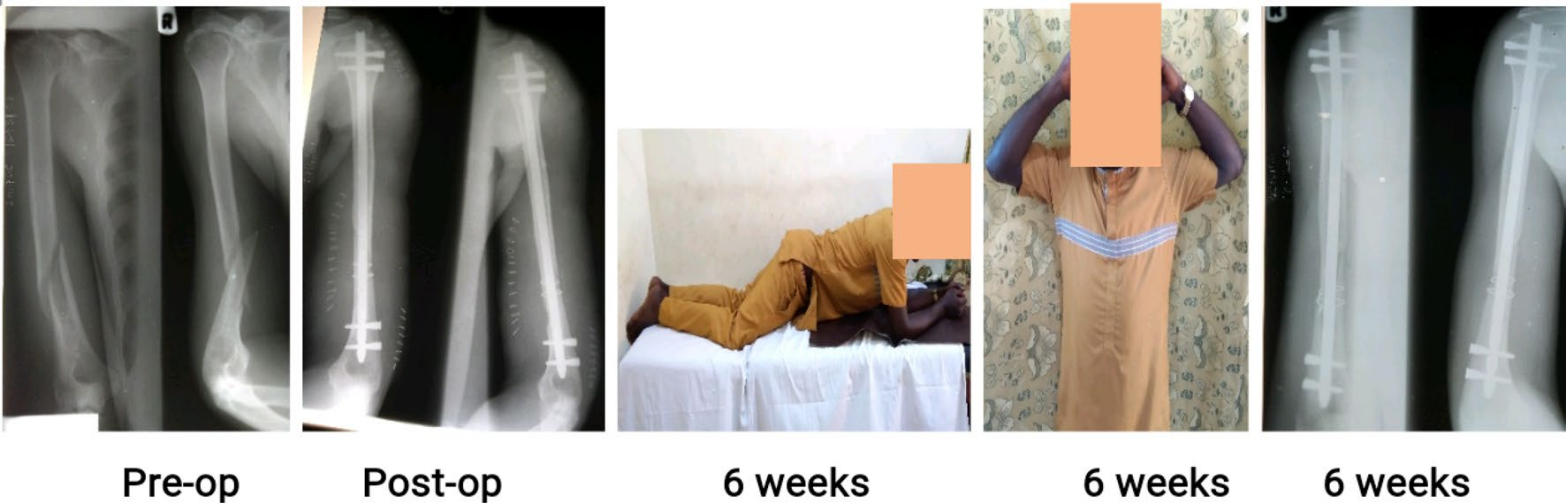
A 35-year old factory worker with atrophic non-union of a 12-A1 fracture nailed 3.52 months post-injury. He was back at work before the 6-week follow-up.

**Figure 4:**
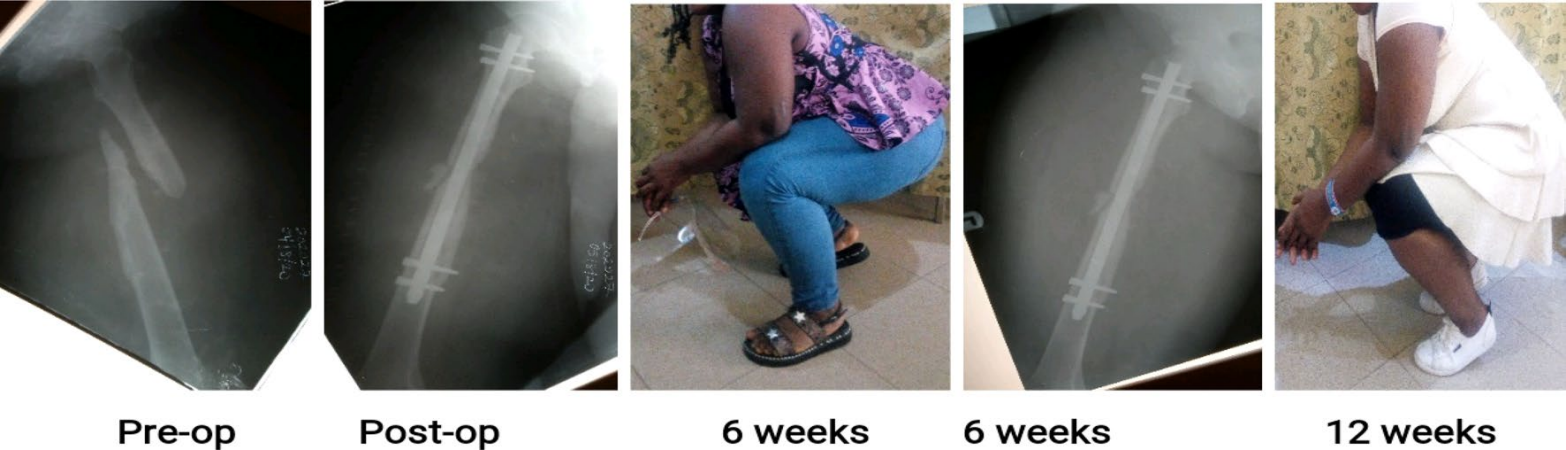
A 41-year old public servant with neglected atrophic non-union of a mid-shaft femur fracture fixed 92.8 months post-injury. She had walked with a limp and a stick before she had the fractured fixed, and was glad to return to work without the limp and the stick immediately after the 6th week follow-up visit, even without radiological union.

**Figure 5:**
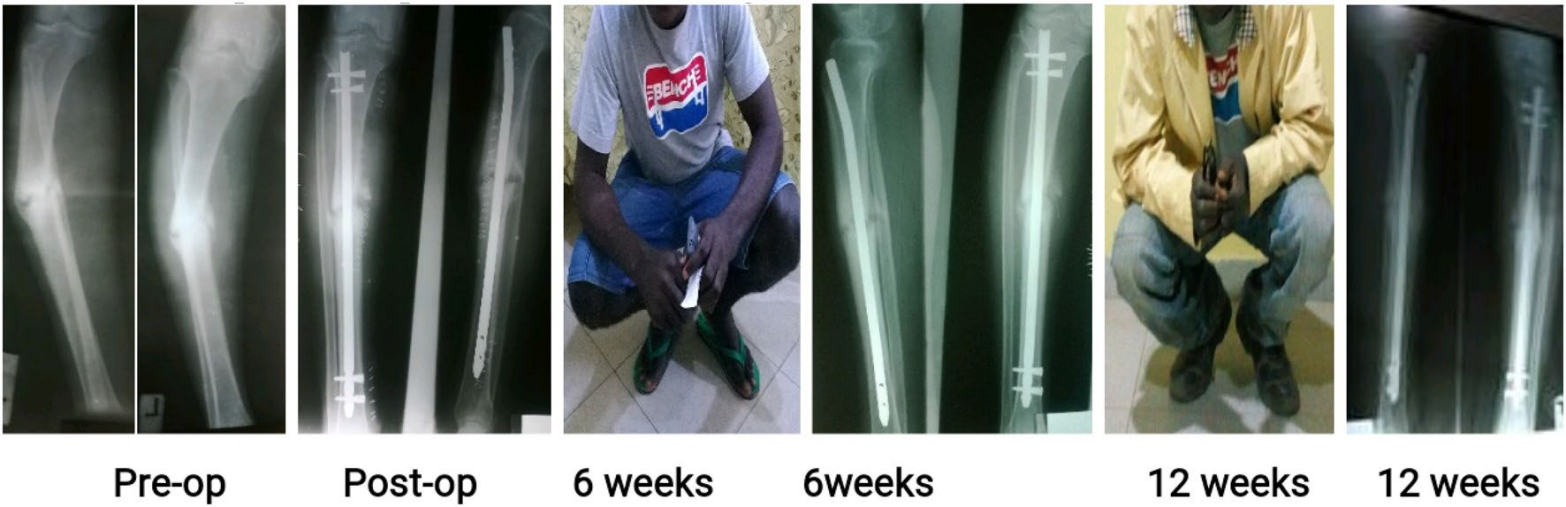
A 34-year old heavy construction machinery operator with hypertrophic non-union of a 42-A3 fracture operated 11.64 months post-injury. He was back at work immediately after the 6th week follow-up visit in spite of absent radiological union.

Figures 3, 4 and 5 are clinical photographs of some patients who returned to work shortly after their fractures were treated, before radiological union occurred.

## Discussion

In many LMICs, people seek fracture care from bonesetters irrespective of the cause, location, or type of fractures, and the patronage cuts across socio-demographic boundaries^9,14^. Our findings (Tables 1, 2 and 3) are in consonance with this previously documented assertions. There were more closed, simple/wedge (AO/OTA type A and B) and diaphyseal fractures than open, multi-fragmentary (type C) and end-segment fractures. This is similar to the pattern reported by earlier studies in our environment^27,28^. Nevertheless, it is also possible that the number of open fractures that eventually presented to us as AFUs was reduced by pre-hospital fatal complications such as septicaemia and tetanus^18,19^ or that many patients with open fractures sought orthodox care of the wound^16^.

Non-union was the most common form of AFUs in our series, and this has been reported by other studies on complications of TBS^10,20^. High energy injuries, the cause of majority of the fractures in our series, are known to predispose to non-unions owing to the accompanying soft tissue disruption^3^. The excessive movement at the fracture site produced by bonesetters’ practice of daily massage could also be responsible^3,9^. With a mean fracture-surgery interval of 21.04 months and a range of 1.12–219.03 months, the fractures in our study were much older than those of similar previous studies^9,10,29^. Since SIGN Fracture Care International started donating implants to our centre, patronage for fracture care has considerably increased, including old fractures which had hitherto been neglected. Many people in our area are often reluctant to seek surgical care in hospitals of neighbouring states due to distance, poverty and unfamiliarity with the hospitals.

In assessing treatment outcome, we placed greater emphasis on the patients’ functional recovery rather than radiological union. Despite the extensive amount of research on finding reliable ways of determining fracture healing, no ‘gold standard’ methods of assessing fracture union currently exist^21^. Hence, clinicians are to draw on multiple assessment modalities that measure or correlate with bone healing^21^. The modalities they draw on, however, is dependent on the ones available and feasible in their practice location/culture. Hence, for our study, the indices employed in the outcome assessment included patients’ abilities to: (i) achieve full WB (ii) squat and smile (S&S) (or do shoulder abduction and external rotation [SAER] for humerus fractures) and, (iii) flex the knee (KF > 90°) or abduct the shoulder (SA > 90°) beyond 90°.

Test for painless WB has remained one of the mainstays of determining fracture union in the clinical setting notwithstanding many advances in fracture union assessment^30^. However, in addition to physician-based clinical and radiological methods, evaluations of fracture healing should also incorporate a patient-centered approach which includes patients’ goals and expectations from the healing process as it relates to their physical and mental functioning^21,31^. Fascinatingly, we observed that the SIGN nail being a solid locked rod, allows early WB and return to pre-injury daily activities even when the fracture has not radiographically healed. Thus some of our patients with simple diaphyseal fractures started unaided ambulation before discharge from the hospital, and by the 12th week follow-up visit, three-quarters or more had achieved FWB, S&S/SAER and KF > 90°/SA > 90°, and had returned to work.

Conversely, Ogunlade et al.who treated similar fractures with plate and screws in an analogous population initially mobilised their patients on non-WB crutches for 6–8 weeks, partial WB when callus was radiographically visible and full WB only when the fracture was “judged to have healed enough”, the whole process taking 3–4 months^9^. Another comparable study by Madu et al. ^11^ reported a better outcome with locked IM nailing than plating of femoral non-unions. Locked IM nailing is known to tolerate early WB and joint motion^1,11^. Hence, immediate WB as tolerated has been recommended for tibial and femoral shaft fractures treated with locked IM nail whereas initial 6–8 weeks of touch-down WB followed by progressive WB is recommended for plate and screws osteosynthesis of similar fractures^5^.

In our environment, most patients seek fracture care to regain the use of their limbs to an extent that allows them return to their pre-injury activities and work. Once this is achieved, they consider their fractures to have healed, and would often self-stop further follow-up irrespective of radiological findings^1,32^. The fact that the S&S test is based on squatting makes it locally relevant—for social, cultural, religious or occupational reasons. Since it can assess the mobility and stability of joints, especially hip and knee, the quality of squatting is said to be a proxy reflection of the functional outcome after fixation of lower limb fracture, particularly in LMICs where other assessment modalities are either expensive or unavailable^31,33^.

We encountered a lower incidence of complications than reported by previous authors who used implants other than locked IM nail: Three humerus fracture cases (15%) had radial nerve palsy which had recovered by the 12th week follow-up, but Madu et al.^11^ and Olasinde et al.^12^ reported 23.5% and 27.3% respectively in patients treated with plate and screws. One tibia fracture had superficial surgical site infection which healed with debridement and antibiotic treatment while three (3.1%) fractures had deep infection for which the implants were removed after the fractures had healed. The infection resolved subsequently.

## Conclusion

Our study has shown the diversity of patients and fracture characteristics that were treated by TBS. It also revealed the forms of consequent AFUs and the valuableness of the SIGN’s solid locked IM nail in salvaging the fractures in a way that permitted early WB. Besides public health education, easy proximate access to efficient orthopaedic implants is a potential preventive public health mechanism to reduce the patronage of bonesetters and consequent complications of TBS. This is because such implants allow for a shorter hospital stay and early return to patients’ pre-injury economic activities and are therefore poverty-mitigating.

Nevertheless, the small number of fractures treated and the descriptive nature of our data analysis are limitations to foregoing conclusion. Further studies involving larger number of AFUs are needed to establish the statistical significance of the findings in this study.

## Data Availability

The datasets generated during and/or analysed during the current study are available from the corresponding author on reasonable request.
